# Lithium-discontinuation-induced treatment refractoriness revisited

**DOI:** 10.1186/s40345-024-00339-6

**Published:** 2024-05-15

**Authors:** Ralph Kupka, Eline Regeer, Annet van Bergen, Leonardo Tondo, Michael Bauer

**Affiliations:** 1 Dept. of Psychiatry, Amsterdam University Medical Center, Vrije Universiteit, Amsterdam Public Health Research Institute, Oldenaller 1, Amsterdam, 1081 HJ Netherlands; 2 Altrecht Institute for Mental Health Care, Utrecht, Netherlands; 3 GGZinGeest Institute for Mental Health Care, Amsterdam, Netherlands; 4 McLean Hospital - Harvard Medical School, Boston, MA USA; 5 Lucio Bini Mood Disorders Center, Cagliari, Italy; 6 Dept. of Psychiatry and Psychotherapy, Faculty of Medicine, Technische Universität Dresden, Dresden, Germany

**Keywords:** Lithium, Discontinuation, Treatment refractoriness, Bipolar disorder

## Abstract

**Background:**

Lithium is effective in the long-term treatment of bipolar disorder. Concerns have been raised about non-responsiveness after discontinuation and resuming previously effective lithium prophylaxis. We reviewed the available literature on this so-called lithium-discontinuation-induced treatment refractoriness (LDITR).

**Results:**

We found 11 case reports and six cohort studies including 403 patients addressing LDITR, and one nation-wide register study providing some additional data on LDITR. Pooling all cohort studies, the percentages of non-responders during re-treatment with lithium ranged from 3.6 to 27.7%, with an average of 17.3%. Non-responsiveness was associated with longer duration of lithium treatment before discontinuation, longer duration of bipolar disorder before start of lithium, faster tapering off lithium, and longer duration of discontinuation.

**Conclusions:**

There may be a subgroup in whom lithium discontinuation-induced treatment refractoriness exists. However, the vast majority of people respond when lithium is restarted. Moreover, it may be necessary to continue lithium beyond the first relapses to restore long-term prophylactic efficacy.

## Background

Lithium is regarded as the first-line prophylactic medication to prevent recurrences of manic and depressive episodes in bipolar disorder (Goes 2023; Severus et al. 2018) and to reduce suicide risk (Baldessarini et al. 2010; Dervic et al. 2023). Although lithium is not effective in all patients with bipolar disorder, it is estimated that about one-third of individuals with bipolar disorder are excellent lithium responders (Rybakowski 2014; Papiol et al. 2022). In addition, in a substantial group of patients lithium significantly improves the course of the illness by reducing the severity of episodes without preventing them altogether (Sportiche et al. 2017). After a few years the question may arise whether, and for how long, lithium needs to be continued. Most treatment guidelines give no clear answer other than the recommendation to continue maintenance pharmacotherapy for a long time, since bipolar disorder is assumed to be a lifelong illness with a high risk of recurrence. Still, there may be various reasons to stop prophylactic medications such as lithium (Öhlund et al. 2018). There is some convincing evidence that if a patient reaches the decision to stop lithium treatment, it should be tapered gradually to prevent early recurrences that may occur after a rapid discontinuation (Baldessarini et al. 2010, 2022). This may also apply to other mood-stabilizing medications (Franks et al. 2008). When this tapering strategy is followed and rebound episodes do not occur in the following months without prophylactic pharmacotherapy, there still will be a persisting risk of future episodes. Many patients who want to stop lithium despite its beneficial effect, e.g. because of side-effects, pregnancy, or the wish to live without any long-term medication after a prolonged illness-free time, ask whether lithium will again be effective if they consider to restart it for renewed prophylactic treatment or should future episodes occur. This concern has increased among both patients and professionals since Post et al. (1992) reported on four patients who discontinued effective long-term lithium treatment, relapsed, and were unresponsive after resumption. They termed this ‘lithium-discontinuation-induced treatment refractoriness’ (hereafter LDITR). Since then, case reports and naturalistic cohort studies of this phenomenon have been published. De Vries et al. (2013) reviewed the evidence for LDITR and found five relevant studies (Baldessarini et al. 1999; Coryell et al. 1998; Koukopoulos et al. 1995; Maj et al. 1995; Tondo et al. 1997), of which three were included in a meta-analysis. They concluded that the available literature at that point did not provide convincing evidence that lithium is less effective when restarted after discontinuation. That review only briefly addressed the published case reports of LDITR. Their conclusion is consistent with our own clinical experience that most lithium-treated patients respond after discontinuation and relapse, even repeatedly. However, as the potential risk of LDITR continues to concern both patients and professionals, the aim of this study is to provide an updated overview of the available case-reports and cohort studies, and to discuss the associated clinical implications.

## Method

We searched the following databases using the terms lithium AND discontinuation AND bipolar disorder: Embase (543 results between 1970 and 2023); PubMed (459; 1968–2023); PsychInfo (285; 1993–2022); and Web of Science (423; 1991–2023). We reviewed all titles and abstracts and included all papers that published original data thatwere relevant to the subject of discontinuation and restarting lithium prophylaxis in adult patients with bipolar disorder, unipolar depression, or schizoaffective disorder. The references of relevant papers were screened for other publications on LDITR. From these papers we retrieved the numbers of patients that did and did not show a favourable response to restarted lithium after discontinuation, and the characteristics of these two groups, if provided. Refractoriness to restarted lithium was defined in various ways throughout the included cohort studies: non-remission of the first recurrent episode despite restarting lithium; occurrence of at least one mood episode in the year after restarting lithium; or a more unfavorable course of illness after restarting lithium when compared to the first lithium treatment in that patient (in one study differentiated in partial responder and non-responder).

## Results

Six case reports with a total of 11 patients and six naturalistic cohort studies including 403 patients were identified. In addition to those originally reviewed by de Vries (2013), we have included two further studies (Cakir et al. 2017; Fornaro et al. 2016) published subsequently. Furthermore, we uncovered a large-scale nationwide health care registry study assessing the efficacy of pharmacotherapy in individuals with bipolar disorder who had previously discontinued lithium (Holm et al. 2022).

### Case reports

**Table 1:** Published cases of lithium-discontinuation-induced treatment refractoriness

Publication	Patient sex, age, diagnosis	Years of stability on Li treatment	Months to relapse after stopping Li	Recurrence after restarting Li	Remarks
Post et al. 2002; Post and Leverich 2008 (case by G.M. Goodwin)	M, 44, BD-I	10	6	Dep - hM - RC	Severe depressions; committed suicide 2 years later on lithium again
Post et al. 1992	F, 43, BD-II	7	18	Dep - hM - RC	Gradual discontinuation; recurrences despite lithium and various other drugs during 3-year follow-up
Post et al. 1992	F, 67, BD-I	5	n.r.	n.r.	Failed to respond once lithium was reintroduced after the emergence of a new episode
Post et al. 1992	F, 34, BD-I	6	n.r.	n.r.	Failed to respond once lithium was reintroduced after the emergence of a new episode
Post et al. 1992	F, 52, BD-I	15	n.r.	n.r.	Failed to respond once lithium was reintroduced after the emergence of a new episode
Bauer 1995	M, 50, BD-I	12	6	Mania - Dep	Refractory to lithium and other medications during 3-year follow-up
Oostervink et al. 2000	M, 66, BD-I	20	36	Dep	Gradual discontinuation; refractory to various medications
Oostervink et al. 2000	M, 60, BD-I	5	1	Mania - Dep - RC	Gradual discontinuation; remitted by adding valproate and tranylcypromine
Oostervink et al. 2000	M, 26, BD-I	5	1	hM - Dep - RC	Gradual discontinuation; remitted by adding carbamazepine
Collumbien 2000	F, n.r., BP-I	9	Some months	Mania	Refractory to lithium and other medications
Appleby et al. 2006	M, 54, BD-I	17	1	Dep - Mania - RC	Total treatment refractoriness

Abbreviations: BD-I = bipolar-I disorder; BD-II = bipolar-II disorder; Dep = depression; F = female; M = male; hM = hypomania; RC = rapid cycling; n.r. = not reported

The 11 individual cases are described in Table 1. These were six men and five women, aged between 26 and 67 years (mean 49.6 ± 13.3), having used lithium successfully over a period ranging from 5 to 20 (mean 10.1 ± 5.3) years. They had been diagnosed with bipolar-I (*n* = 10) or bipolar-II (*n* = 1) disorder. As was reported in eight cases, the first recurrence occurred between one month (*n* = 3) and 36 months (mean 9.0 ± 12.3 months), and was depressive (*n* = 4) or (hypo)manic (*n* = 4); in five of these eight cases the first recurrence was followed by a rapid cycling course. Of three cases no further details about the course after restarting lithium were given. Most patients were neither responsive to lithium nor to various other mood-stabilizing drugs. Since the description of the cases is highly variable, it is difficult to draw common characteristics of these patients. In their report, Post et al. (1992) also mentioned that among 66 patients who had been referred to NIMH with refractory bipolar illness after being treated for at least one year with lithium, seven (10.6%) had a clear-cut LDITR and two (3.0%) had a possible LDITR.

### Cohort studies

**Table 2:** Cohort studies on lithium-discontinuation-induced treatment refractoriness

Publication	*N*	Diagnosis	Years of Li treatment before discontinuation (mean)	Years of follow-up after restarting Li (mean)	Outcome after restarting lithium
Koukopoulos et al. 1995	89	BD-I and BD-II	8.8	[> 1]	13 (15%) were non-responders after restarting
Maj et al. 1995	54	BD-I	5.9	1	10 (19%) were non-responders after restarting
Tondo et al. 1997*	86	BD-I and BD-II	4.6	4.4	25 (29%) were episode-free after initial treatment 20 (23%) were episode-free after restarting
Coryell et al. 1998	28	RDC mania or SzAff mania	n.r.	3	13 (45%) had ≥ 1 relapse after first trial 9 (33%) had ≥ 1 relapse after restarting
Baldessarini et al. 1999*	130	BD-I and BD-II	4.2	4.0	77% was ≥ 90% of time well after initial treatment; and of these, 37 (28.5%) were episode-free 67% was ≥ 90% of time well after restarting; and of these, 26 (20%) were episode-free
Fornaro et al. 2016	37	BD-I	4.0	1.15	4 (11%) were non-responders after restarting
Cakir et al. 2017	65	BD-I and BD-II	4.6	4.0	47 (72%) were equally responsive after restarting 9 (14%) were less responsive after restarting 9 (14%) were non-responsive after restarting

Abbreviations: BD-I = bipolar I disorder; BD-II = bipolar II disorder; RDC = Research Diagnostic Criteria; n.r. = not reported; SzAff = schizoaffective.

* Same cohort (extended and updated in 1999)

A total of 403 patients participated in the reported six studies. They had mainly been diagnosed with bipolair I or II disorder and had been on lithium for an average of 5.4 ± 1.8 years before discontinuation. The mean time of follow-up after re-starting lithium was 2.7 ± 1.6 years.

Koukopoulos et al. (1995) described various aspects of the long-term prophylaxis of affective disorders. Of the total cohort of 375 patients, 221 had bipolar-I disorder, 122 bipolar-II, and 32 unipolar depression. All patients were treated with lithium for at least five years. Of these, 110 (29.3%) stopped lithium once or several times because feeling well, experiencing minor side effects while being euthymic, or pregnancy. In most patients, euthymic mood had been present for more than one year. During follow-up, recurrence occurred in 89 (23.7%), after which lithium was restarted. Of these patients, 13 (14.6%, seven women and seven men) showed lack of responsiveness despite eight (61.5%) had been being completely well and five (38.5%) substantially better on lithium for 1–11 years (mean 7.8). The nonresponsiveness lasted 2–7 years (mean 4.2), and included nonresponsiveness to other medications and ECT as well.

Maj et al. (1995) included 24 men and 30 women with bipolar I disorder who had discontinued lithium for reasons other than recurrence of illness or serious side effects: feeling cured (*n* = 26, 48.1%), somatic side effects (*n* = 14, 25.9%), unwilling of taking medications (*n* = 6, 11.1%), loss of energy or productivity (*n* = 5, 9.3%), or decision to become pregnant (*n* = 3, 5.6%). Lithium prophylaxis was restarted after a manic or major depressive episode occurred, which happened 1–58 months (mean 11.4) after discontinuation. ‘Responders’ (*n* = 44, 81.5%) were patients who did not have an episode in the first year after restarting lithium, ‘non-responders’ (*n* = 10, 18.5%) experienced at least one episode. The only significant difference between the two groups was a longer duration of lithium treatment before discontinuation in the ‘non-responders’ (8.4 versus 5.4 years). At longer follow-up, two (20%) of the 10 non-responders had multiple recurrences but then stabilized on continued lithium during the next two years. The authors concluded that nonresponse to reinstituted prophylaxis should be considered as a risk of interrupting an effective long-term lithium treatment. They also suggested that the refractory state may be reversible in some patients, making it worthwhile to continue lithium beyond the first recurrence.

Coryell et al. (1998) included 10 men and 18 women who had been previously diagnosed with RDC mania or schizoaffective disorder, manic type, had recovered from the index episode on lithium, and remained on lithium for at least two years with a good response. All patients discontinued lithium, for reasons not reported, for a period of 2-303 weeks (mean 50.2) before they had a first prospectively observed recurrence while being off lithium. When lithium was reinstituted, 27 (96.4%) recovered. The median time to recovery (4.0 weeks) did not differ between the index episode and the first prospectively observed episode during the second lithium treatment. Also, recurrences during a three-year follow-up while being on lithium after the index episode occurred in 28 (45.0%) cases and in 27 (32.9%), with no significant differences. Likewise, there was no difference in the additional use of antipsychotics or antidepressants in the two periods. The authors concluded that these findings provided no evidence that discontinuation of lithium lead to treatment resistance after resuming lithium.

Baldessarini et al. (1999), extending and updating the sample previously published by Tondo et al. (1997), included 130 patients with bipolar-I or -II disorder who had received a first treatment with lithium (with a mean duration of 4.2 years), unselected for the quality of the response to lithium. Patients who discontinued lithium because of an emerging mood episode (hypomanic or manic) were excluded. Included patients had discontinued lithium for 1.9 ± 2.3 years and subsequently had a second treatment for an average of 4.0 years. When comparing the course of illness in all patients during the first and the second trial, the only significant difference was a shorter time in depression during the first trial (10.7 ± 17.0%) than during the second trial (15.2 ± 21.2%). During the first trial, more patients remained well ≥ 90% of the time (77.0% vs. 66.9%; not significant). During the first trial, 37 (28.5%) patients had no recurrences; of these 37 patients, only 21 (57%) were completely episode-free during the second trial. Of the total sample, 26 (20%) were completely episode-free in the second trial compared to 37 (28.5%) in the first trial (no significant difference). Use of additional antipsychotics or antidepressants was not significantly different during the first (51.5%) versus the second (42.9%) trial. Based on these findings, these authors concluded that the average benefits of lithium with respect to overall morbidity in an unselected clinical sample are only slightly less during re-treatment with lithium after discontinuation and subsequent recurrence. They also suggested that one should not regard a single recurrent episode after restarting the treatment as an indication of (prophylactic) non-response.

In the first of the two studies that were not included in De Vries’ review and meta-analysis, Fornaro et al. (2016) retrospectively reviewed the charts of 20 men and 17 women, median age 52 years, with bipolar-I disorder, who had been stable on lithium for a median duration of four years before they stopped long-term lithium maintenance treatment for a median duration of 5.5 years. All patients subsequently restarted lithium and were observed for a median duration of 1.2 years. Of these, 33 patients (89.2%) again responded to lithium, while four (10.8%) failed to respond after restarting lithium. Non-responders only differed from re-responders in that they had a longer duration of bipolar illness before the first treatment with lithium (median 8.5 versus 3 years), a longer duration of discontinuation (median 5.5 versus 2 years), and a faster tapering off lithium (median 1 versus 7 days).

The second study, by Cakir et al. (2017), included 29 men and 36 women with bipolar-I (*n* = 47, 72.3%) or -II (*n* = 18, 27.7%) disorder that had been treated with lithium for a duration of 24–156 months (mean 55.2 months). Of these, 19 (29.2%) had an excellent response to lithium, defined as ‘no major or minor mood episodes during treatment’, and 46 (70.8%) had a partial response, defined as ‘reduction in frequency, duration, or severity of mood episodes compared with the pre-lithium period’. All patients discontinued lithium (reasons not reported) for 4-129 weeks (mean 21.9 weeks), during which period 15 patients (23.1%) had a manic (*n* = 6), mixed (*n* = 4), hypomanic (*n* = 3), or depressive (*n* = 2) episode. All 65 patients restarted lithium and were treated for 28–90 months (mean 47.9 months). Reasons for restarting lithium were not mentioned, but it is of notice that only a minority of patients (15/65) restarted because of a recurrence during the off-lithium period. In the second period, seven patients (10.8%) had an excellent response, 49 (75.4%) had a partial response, and nine (13.8%) had a poor response, defined as ‘lack of reduction in frequency, duration, or severity of mood episodes’. When compared to their first period on lithium, 47 patients (72.3%) had no change in excellent (*n* = 7) or partial (*n* = 40) responsiveness, nine (13.8%) changed from excellent responder to partial responder, three (4.6%) changed from excellent responder to non-responder, and six (9.2%) changed from partial responder to non-responder. Overall, there was a partial or complete loss of responsiveness in 18 patients (27.7%). When these 18 patients with decreased responsiveness were compared with the 47 patients with unchanged responsiveness, the groups were largely similar in demographic and illness characteristics, apart from a significant longer mean duration of lithium discontinuation (8.2 versus 3.9 months) and more often relapsing during the discontinuation period (61.1% versus 8.5%). The authors concluded that although most lithium responders continued to maintain their responsiveness after discontinuation and restarting lithium, a quarter showed a decreased response.

### Nationwide cohort study

Holm et al. (2022) investigated the effectiveness of mood stabilizers (including lithium) and antipsychotics in the prevention of hospitalization and treatment failure after discontinuation of lithium, using data from nationwide health-care registers in Finland between 1987 and 2018. They identified *n* = 4052 individuals aged 15–65 (mean 47.2 ± 13.7 years) and 54% women, who had discontinued lithium after using it for at least one year (median 2.7 years). The authors state that their sample consisted of long-term lithium users with a relativelysuccessful response who tolerated lithium well. Lithium was discontinued for other reasons than death, hospitalization, or end of follow-up. Medications that were studied after lithium discontinuation included mood stabilizers (lithium, valproate, lamotrigine, and carbamazepine), oral antipsychotics (quetiapine, olanzapine, aripiprazole, levomepromazine, risperidone, and chlorprothixene), and long-acting injectable antipsychotics (LAIs). Treatment failure was defined as psychiatric hospitalization, death, or a change in the use of mood stabilizing or antipsychotic medications (i.e., switch, discontinuation, or add-on). The latter was the most common reason for treatment failure. The median follow-up duration from lithium discontinuation until hospitalization, death, or end of study was 8.9 ± 6.2 years. The most commonly used medications under investigation during this follow-up were: antidepressants (61%), quetiapine (57%), benzodiazepines (50%), and lithium (37%). Among mood stabilizing monotherapies, restarting lithium was associated with the lowest risk of subsequent treatment failure. LAIs and valproate were effective in preventing hospitalization in this sample, in contrast to quetiapine and olanzapine. Although the purpose of this study was not to investigate LDITR, the results suggest that in a large proportion of patients lithium was again effective when reinstituted after discontinuation.

## Discussion

We reviewed the currently available evidence from case reports and cohort studies on whether discontinuing an effective prophylactic treatment with lithium in patients with bipolar disorder leads to refractoriness when restarting the treatment after a first recurrence of depression or mania. Obviously, although case-reports can draw attention to a particular clinically relevant phenomenon, given their anedoctal nature they do not allow to draw firm conclusions. The published cases of LDITR are highly heterogeneous, as is the course of illness after restarting lithium, and the medications used to achieve remission, whether or not successful. Moreover, we could easily write numerous case reports of patients who do respond to lithium after stopping and restarting, even multiple times. Thus, findings from longitudinal cohort studies may provide more significant insights.

Pooling all cohort studies, our review found that on average 17.3% (62/359) of participants did not respond to lithium re-treatment [Koukopoulos: 13/89 (14.6%); Maj: 10/54 (18.5%); Coryell: 1/28 (3.6%); Tondo-Baldessarini: 16/86 (18.6%); Fornaro: 4/37 (10.8%); Cakir: 18/65 (27.7%)].

This finding aligns with the original research conducted by Post and colleagues (Post et al. 1992; Post and Leverich 2008), who concluded that LDITR is a relatively rare phenomenon affecting approximately 10–15% of patients.

Attempting to identify the mechanisms behind this phenomenon, it remains uncertain whether the non-responsive group is attributable to discontinuation of lithium or to reduced pharmacological treatment efficacy as a consequence of illness progression. Post et al. (1992) suggested that it could pose a risk to individuals with a highly recurrent condition but is less prevalent in a broader group of patients with BD characterized by a milder and less recurrent ailment, which is a conclusion similar to the nationwide investigation by Holm et al. (2022). A review by Joyce et al. (2016) suggests that treating bipolar disorder in its early stages is more effective than in its later stages.

In a review of LDITR, Post (2012) suggested three possible explanations, which may interact: (1) the occurrence of new episodes (off lithium) may change the subsequent course of illness by the general phenomenon of episode sensitization; (2) given the neuroprotective and neurotrophic effects of lithium, discontinuation may further increase the neuropathology of the illness; and (3) new episodes in the absence of these neuroprotective effects of lithium may be more pernicious and transform the illness in a more treatment-refractory state. There is an increasing body of pre-clinical and clinical research indicating that lithium administration may have long-term neurotrophic and protective effects (Puglisi-Allegra et al. 2021). Consequently, cessation of lithium treatment may result in an interruption of the latter effects and proceed toward a more unfavourable disease course (Cakir et al. 2017). Certain genetic mechanisms have been linked with lithium responsiveness and might also be correlated with increased vulnerability to treatment resistance subsequent to its discontinuation (Cakir et al. 2017).

The following findings from the studies described in this review are suggestive of an effect of disease progression and perhaps not a specific effect of lithium discontinuation. The ‘non responders’ in the Koukopoulous et al. (1995) study indeed showed lack of responsiveness lasting for 2–7 years (mean 4.2), and included non-responsiveness to other medications and ECT as well. The study by Maj et al. (1995), which included only ‘excellent responders’ showed that the duration of lithium treatment before discontinuation was significantly longer in non-responders (8.4 versus 5.4 years). Non-responders in the Fornaro et al. (2016) study showed a longer time of untreated BD before first ever lithium treatment (8.5 years compared with 3 years in responders), and also a longer duration of discontinuation before restarting lithium (5.5 years for nonresponders compared with 2 years in responders). Evidence of the influence of disease progression was also found in the Cakir et al. (2017) study: the group with the decreased responsiveness relapsed more often during the discontinuation period than the group with unchanged responsiveness (61.1% vs. 8.1%).

In summary, our findings raise questions about the nature of the increased risk of relapse following discontinuation of lithium treatment in these patients. Apart from a natural course of disease progression (independent from lithium discontinuation), which may be further accelerated by new episodes after lithium discontinuation (episode sensitization), there may be an additional risk by removing the neuroprotective and neurotrophic effects of lithium. The resulting treatment resistance may not only be lithium specific (Joyce et al. 2016).

### Clinical implications

For clinical practice, it would be helpful to identy patients who are more prone to lithium non-response after discontinuation and re-initiation. By reviewing the cohort studies, we found only a few characteristics, such as longer duration of lithium treatment before discontinuation (Maj et al. 1995), longer duration of bipolar disorder before starting lithium, faster tapering of lithium (Fornaro et al. 2016), and longer duration of discontinuation (Cakir et al. 2017; Fornaro et al. 2016). The decision to discontinue prophylactic lithium should be the result of a shared decision-making process with the patient, caregiver and professional, in which the risks (pros and cons) are weighed. In the event of discontinuation, lithium should be tapered slowly, as shown in the study by Fornaro et al. (2016), in which non-responders were tapered off lithium more rapidly. A study of Baldessarini et al. (2022) also showed a higher risk of relapse after rapid discontinuation (1–15 days) than after gradual discontinuation (more than two weeks). If lithium is restarted because of a recurrence, it is advisable to continue for a longer period, even if there is no immediate effect. The study by Maj et al. (1995) showed that the refractory state may be reversible in some patients. There may also be a partial response (attenuated response) which was found in the study of Cakir et al. (2017). Since lithium is primarily a prophylactic treatment in BD, in case of previous efficacy, we would advise to continue lithium after restarting, even in case of initial non-response, add guideline-recommended treatment options for the acute treatment of the manic or depressive episode, and in the longer term make a renewed attempt for lithium monotherapy as maintenance treatment.

### Limitations

First, published cases studies have a risk of emphasizing a relatively rare phenomenon which may not be applicable to the large group of lithium-treated patients with BD. An second limitation of this review is the heterogeneity of the study populations included in the cohort studies and the differences in naturalistic study design and definition of outcomes. Therefore, we decided not to attempt a further meta-analysis of the results. Not all studies in our review (Cakir et al. 2017; Coryell et al. 1998) reported the reasons for discontinuation. If the reason for discontinuation is not well defined, the possibility of reduced efficacy of lithium prior to discontinuation cannot be completely excluded. Furthermore, details of the course of the illness (e.g. number of episodes before discontinuation) are not reported in all studies, although this could influence treatment response (Joyce et al. 2016). Furthermore, the results of the study of Baldessarini (1999) suggest that a single relapse after restart should not be taken as an indication of (prophylactic) non-response. Most studies include excellent responders, which may increase the finding of discontinuation-induced refractoriness (de Vries et al. 2013; Cakir et al. 2017).

## Conclusions

A request from a patient with BD to discontinue lithium prophylaxis should not be dismissed by a warning that lithium will not work again after restarting. After a careful review of the illness history and the effectiveness of medications used, and evaluation of the rationale for discontinuation, potential risk factors for subsequent non-response should be discussed. Approximately one in six patients may be confronted with (initial) lithium discontinuation-induced treatment refractoriness. By reviewing the cohort studies, we found only a few associated characteristics, such as longer duration of lithium treatment before discontinuation, longer duration of bipolar disorder before starting lithium, faster tapering of lithium, and longer duration of discontinuation. Still, the vast majority of patients respond when lithium is restarted, and if not immediately in the acute phase, lithium prophylaxis may again prove efficacious in the longer term. Further research is required to gain a better understanding of the relatively rare occurrence of so-called “lithium-discontinuation-induced treatment refractoriness”.

## Data Availability

No datasets were generated or analysed during the current study.

## Abbreviations

LDITR: Lithium-Discontinuation-Indiced Treatment Refractoriness
